# Callose plug deposition patterns vary in pollen tubes of *Arabidopsis thaliana* ecotypes and tomato species

**DOI:** 10.1186/1471-2229-12-178

**Published:** 2012-10-03

**Authors:** Peng Qin, Dylan Ting, Andrew Shieh, Sheila McCormick

**Affiliations:** 1 Plant Gene Expression Center, U.S. Department of Agriculture/Agricultural Research Service and Department of Plant and Microbial Biology, University of California at Berkeley, Albany, CA, 94710, USA; 2 Current address: Rice Research Institute of Sichuan Agricultural University, Chengdu Wenjiang, Sichuan, 611130, China

**Keywords:** Callose plugs, Pollen tube growth, Sperm

## Abstract

**Background:**

The pollen grain contains the male gametophyte that extends a pollen tube that grows through female tissues in order to deliver sperm to the embryo sac for double fertilization. Growing pollen tubes form periodic callose plugs that are thought to block off the older parts of the tube and maintain the cytoplasm near the growing tip. The morphology of callose plugs and the patterns of their deposition were previously shown to vary among species, but variation within a species had not been examined. We therefore systematically examined callose plug deposition in *Arabidopsis thaliana* ecotypes, tested for heritability using reciprocal crosses between ecotypes that had differing deposition patterns, and investigated the relationship between callose plugs and pollen tube growth rate. We also surveyed callose plug deposition patterns in different species of tomato.

**Results:**

We used in vitro grown pollen tubes of 14 different *A. thaliana* ecotypes and measured the distance from the pollen grain pore to the first callose plug (termed first interval). This distance varied among *Arabidopsis* ecotypes and in some cases even within an ecotype. Pollen tubes without a callose plug were shorter than those with a callose plug, and tubes with a callose plug near the grain were, on average, longer than those with the first callose plug farther from the grain. Variations in the first callose plug position were also observed between different species of tomato.

**Conclusions:**

We showed that the position of the first callose plug varied among *Arabidopsis* ecotypes and in tomato species, and that callose plug deposition patterns were heritable. These findings lay a foundation for mapping genes that regulate callose plug deposition or that determine pollen tube length or growth rate.

## Background

In angiosperms, the pollen grain contains the male gametophyte. The male gametophyte extends a pollen tube that grows through female tissues in order to deliver sperm to the embryo sac for double fertilization. Callose, a β 1,3-glucan, is the major component of the pollen tube cell wall [1]. Callose can be visualized by staining with decolorized aniline blue [2]. Growing pollen tubes form periodic callose plugs that are thought to block off the older parts of the tube and maintain the cytoplasm near the growing tip [3]. Callose plugs exist in pollen tubes of all flowering plants, although their morphology and the pattern of callose plug deposition varies among species [4]. Callose plugs were proposed as a critical novelty for accelerated pollen tube growth during angiosperm evolution [5], as angiosperm pollen tubes have callosic walls and callose plugs, whereas callose plugs are absent in pollen tubes of gymnosperms.

Callose plug deposition has been correlated with pollen tube growth rate. For example, the number of callose plugs was used as an indicator of pollen tube growth rate in *Hibiscus moscheutos*[6,7]. In tomato, antisense lines for the pollen receptor kinase LePRK2 showed abnormal callose plug deposition and slower pollen tube growth than in wild type [8]. LeSHY is a protein that interacts with LePRK2; in *Petunia*, antisense lines for *SHY* showed no callose plugs in pollen tubes, and nearly all the pollen tubes failed to reach ovules [9]. However, in *Arabidopsis*, one report [10] questioned a need for callose plugs, as pollen tubes carrying a mutant allele of callose synthase 5, *cals5-3,* had no callose plugs, but grew normally in vitro and in vivo. Seed set on *cals5-3* homozygous plants was normal, although transmission of the *cal5-3* allele was slightly reduced in heterozygotes (when in competition with WT pollen tubes).

*Arabidopsis* has been used as a model plant for genetic studies. Many ecotypes have been used to investigate genetic variation in numerous physiological processes [11], and recombinant inbred lines and doubled haploid populations have been used to map genes underlying such variation [12,13]. Although callose plug morphology differs in different species [4], to our knowledge variation within a species has not been examined. During the course of other experiments, we noticed that pollen tubes of the *Arabidopsis thaliana* ecotypes L*er* and Columbia (Col) appeared to vary in the position of the first callose plug; Columbia had the callose plug near the grain, while in L*er* the callose plug was farther away. We therefore decided to systematically examine callose plug position in many *Arabidopsis* ecotypes, and in tomato species. Here we document variation of callose plug deposition in *Arabidopsis* ecotypes and in tomato species [14] and demonstrate that there is a relationship between callose plug deposition and pollen tube growth.

## Results and discussion

### In vitro pollen germination is robust and reproducible in many ecotypes

**Figure 1:**
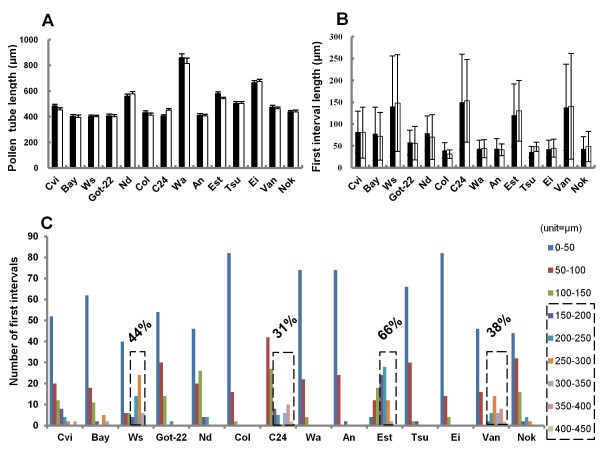
**Pollen tube length and the first callose plug position vary in *Arabidopsis *.** (**A**) Average pollen tube length from 50 randomly selected pollen tubes after 6h pollen germination. Two independent experiments (black and white bars). An unpaired *t* test was performed with two independent experiments. Error bars, SEM, *P*<0.5. (**B**) Average length of the first interval from 50 randomly selected pollen tubes after 6h pollen germination. Two independent experiments (black and white bars). Error bars, SD. (**C**) Distribution of the first intervals pooled from two independent experiments according to the interval length. 150μm was used as the criterion to group those intervals. The intervals in dashed boxes are those ≥150 μm, and the proportion above the dashed box represents the percentage.

We first investigated whether the in vitro pollen germination protocol developed in our lab for the *Arabidopsis thaliana* Columbia ecotype could be used for other ecotypes [15]. For consistency, we always used 2–3 freshly opened flowers for germination assays. After 6 hours germination, we added decolorized aniline blue to visualize callose plugs, transferred the slides to 4°C to stop tube growth, and photographed slides on which most pollen grains had germinated and pollen tubes appeared healthy. All but one of the ecotypes tested formed pollen tubes and callose plugs. For measurements, we selected pollen tubes for which it was easy to trace the length of the pollen tube from grain to tip (it was more difficult to carry out such measurements if the pollen tubes were in an area of the slide with many pollen tubes that were tangled together). An unpaired *t* test showed that the lengths of fifty such randomly selected healthy pollen tubes of each ecotype were not significantly different, in two independent experiments (Figure 1A). We therefore concluded that in vitro pollen germination and tube growth within each ecotype was reproducible. Pollen of the Shahdara ecotype germinated very poorly in this medium, and the few pollen tubes that did form had abnormal morphologies. It is possible that a germination medium suitable for Shahdara could be devised, but we excluded it from further experiments. Although we had initially noticed a difference between the position of callose plugs in Col and L*er*, we did not use L*er* in these experiments because its germination percentage was considerably lower than in other ecotypes.

### The first callose plug position varies among ecotypes in *Arabidopsis*

After 6 hours of germination, we measured the first interval length (distance from the pollen grain pore to the first callose plug) of 50 randomly selected pollen tubes in 14 ecotypes. We used the 6 hour time point in order to ensure that all the pollen grains had had ample time to grow a tube, as germination initiation is not synchronous [15]. Within an ecotype, the average lengths of the first interval in two independent experiments were reproducible (Figure 1B) but the average lengths were different in different ecotypes. In about 50% of the ecotypes, the average length of the first interval was around 50μm, indicating that the first callose plug was consistently close to the pollen grain, but in the Ws, C24, Est, and Van ecotypes, the average first interval length was longer, and the standard deviations were extremely large. Large standard deviations were also observed in Cvi, Bay, Nd and Nok.

To further investigate the differences of the first callose plug position in all ecotypes, we pooled together 100 first intervals from two independent experiments and grouped them according to the length of first interval (Figure 1C). There was a wide distribution of first interval lengths among the 14 ecotypes, ranging from 11μm to 394μm. We arbitrarily divided pollen tubes into two groups. In the first group the first interval was <150μm, i.e. the first callose plugs were close to the grain; in the second group the first interval was ≥150μm, i.e. the first callose plugs were farther away from the grain. All ecotypes had some pollen tubes with the first interval <150μm, but some ecotypes also had pollen tubes with the first interval ≥150μm. The portion of pollen tubes with the first interval ≥150μm varied from 0% (e.g. Col) to 66% (e.g. Est) among these 14 ecotypes. We further subdivided this group; if the portion of pollen tubes with the first interval ≥150μm in one ecotype was lower than 20%, we treated that ecotype as having one pattern of first callose plug deposition, i.e. with the first callose plug close to the pollen grain. However, if the portion of pollen tubes with the first interval ≥150μm in one ecotype was higher than 20%, we defined that ecotype as having two deposition patterns. After applying this criterion, Ws, C24, Est and Van had two patterns for first callose plug position (Figure 1C). Thus the first callose plug position not only varied between different ecotypes, but also varied within certain ecotypes.

**Figure 2:**
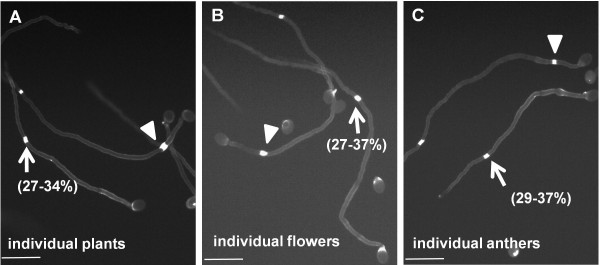
**Variation of the first callose plug position from individual C24 plants, flowers and anthers.** Variation of the first callose plug in one of 6 individual plants (**A**), in one of 6 flowers of one plant (**B**) and one of 6 anthers in one flower (**C**). The percentages represents the proportion of pollen tubes with the first interval ≥150μm. Arrows indicate a first callose plug with the first interval ≥150μm. Arrowheads indicate a first callose plug with the first interval <150μm. Bars = 100μm.

Ecotypes are presumed to be uniform, so this variation of first callose plug position in an ecotype was unexpected. To test whether seed contamination might explain this variation, we used C24. We assayed pollen from 12 individual plants after 6 hours of pollen germination; each showed variation in first callose plug position, and among 12 plants the portions of pollen tubes with the first interval ≥150μm ranged from 27-34% (Figure 2A), similar to the results shown in Figure 1C, suggesting that the variation was not due to seed contamination. We further checked pollen from 6 individual flowers or from individual anthers, each of which still showed variation in the first callose plug position and had similar proportions of pollen tubes with the first interval ≥150μm (Figure 2B and C), indicating that variation of callose plug position in C24 was inherent. Such variation in one ecotype might be due to phenotypic noise [16,17].

### Patterns of callose plug deposition are heritable

**Figure 3:**
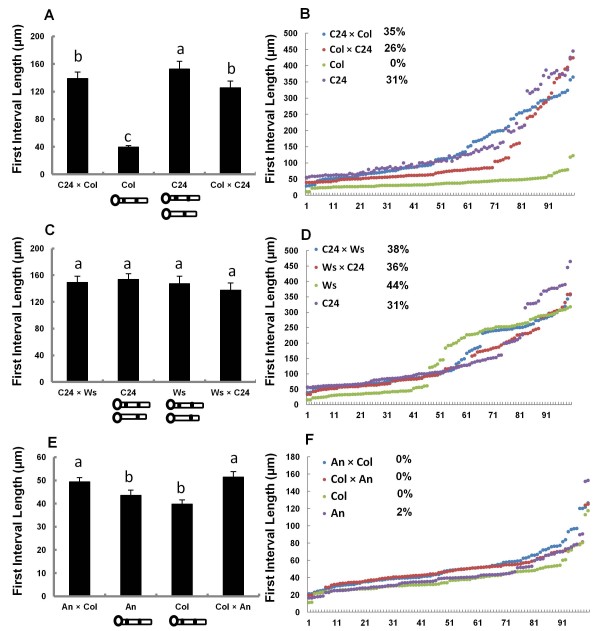
**First callose plug position in parents and F1 progeny of reciprocal crosses.** (**A**), (**C**) and (**E**) show average first interval length of parents and F1s of reciprocal crosses. Comparisons between Col, C24 and their F1 progeny are shown in (**A**); comparisons between Ws, C24 and their F1 are shown in (**C**); comparisons between Col, An and their F1 are shown in (**E**). (**B**), (**D**) and (**F**) show distribution of the first interval length of parents and F1s of reciprocal crosses. Comparison between Col, C24 and their F1 (**B**); comparison between Ws, C24 and their F1 (D); comparison between Col, An and their F1 (F). The percentages in (**B**), (**D**) and (**F**) represent the proportion of pollen tubes with the first interval ≥150μm. ANOVA and multiple comparisons with LSD (least significant difference) were performed. Error bars in (**A**), (**B**) and (**E**) are SEM. Letters indicate significant differences at *P*<0.5. Values followed by the same letter are not significantly different.

To test whether variation of first callose plug position between ecotypes was heritable, we performed reciprocal crosses between ecotypes with different patterns for the first callose plug position. C24 has two patterns while Col has only one pattern (Figure 1C). The average length of 100 first intervals in the two F1s was intermediate of the parental average lengths (Figure 3A). However, the distributions of the first interval length of the two F1s was similar to that of C24, and moreover the proportion of pollen tubes of the two F1s with the first intervals ≥150μm was larger than 20% and thus similar to that of C24 (Figure 3B). This indicated that the two F1s had two patterns of first callose plug position, as did C24. This suggested that the pattern of the first callose plug position was heritable and that having two patterns was dominant. The similar first interval lengths in the two F1s indicated that there were no parental effects on callose plug position. Like C24, Ws has two patterns of first callose plug position (Figure 1B). We therefore reciprocally crossed Ws and C24 to investigate how the two patterns of first callose plug position were inherited in the F1s. The average first interval lengths in the two F1s were not significantly different from those of the parents (Figure 3C), moreover, the distributions of the first interval length in the two F1s were also similar to those of the parents. The proportion of pollen tubes with the first interval ≥150μm were larger than 20% (Figure 3D), which indicated that the two F1s had two deposition patterns. We used reciprocal crosses between Col and An to investigate how the first callose plug position was inherited when the parents only had one pattern for the position of the first callose plug. The distributions of the first interval length in the two F1s were similar to those of the parents (Figure 3F) and no pollen tubes with the first interval ≥150μm were observed in either F1, suggesting that these F1s had one deposition pattern, as did the parents. However, the average lengths of the first intervals in the two Col × An F1s were significantly longer than those of the parents (Figure 3E). Together, these results suggested that variation of the first callose plug position in one ecotype was controlled by genetics, and that the trait of two patterns of deposition of the first callose plug was dominant. The dominance was unexpected, as we predicted that callose plug position would be controlled by the gametophyte, as callose plugs are deposited in pollen tubes after pollen germination. Thus in a gametophytic control scenario and assuming that one gene controls the trait, we expected that 50% of the pollen tubes would have one pattern of first callose plug deposition and 50% of the pollen tubes would have two patterns. C24 had 31% pollen tubes that had long first intervals, therefore only 15% of the F1 pollen tubes were expected to have a long first interval. However, the percentages of pollen tubes with long intervals in F1s from the Col × C24 reciprocal crosses were 35% and 26%, much more than the expected 15%. This did not support that this trait was controlled by a single gametophytic gene. If the trait was controlled by multiple genes, a range of intermediate phenotypes between both parental phenotypes might be expected, but that was not the case. Dominance therefore is most consistent with sporophytic control; however, we don’t understand how the sporophyte would contribute to this phenotype. Before attempting to genetically map the responsible gene(s), it will be important to consider other parameters that might influence this trait, such as sporophytically-derived pollen wall proteins, or paternal provisioning.

### Pollen tube length variability in parents and F1 hybrids

**Figure 4:**
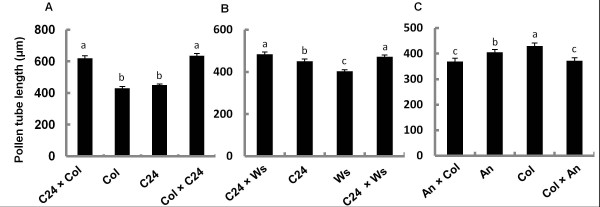
**Pollen tube length in parents and F1 progeny.** (**A**), (**B**) and (**C**) show average pollen tube length of parents and F1s generated from reciprocal crosses. Comparisons between Col, C24 and their F1 progeny are shown in (**A**); comparisons between Ws, C24 and their F1 progeny are shown in (**B**); comparisons between Col, An and their F1 progeny are shown in (**C**). ANOVA and multiple comparisons with LSD were performed. Error bars are SEM. Letters indicate significant differences at *P*<0.5. Values followed by the same letter are not significantly different.

The average length of the pollen tubes varied in different ecotypes after 6h pollen germination (Figure 1A). In most ecotypes, the tube length was around 400μm, but in Wa and Ei they were longer, 860μm and 665μm, respectively. This implied that Wa and Ei pollen tubes grew much faster than those of other ecotypes. When measuring the patterns of callose plug deposition in parents and F1 hybrids (Figure 3), we also noticed that the pollen tube lengths varied in tested F1 hybrids, but there was no difference in reciprocal crosses (Figure 4A, B and C). The pollen tubes of F1s generated from crosses of C24 and Col were significantly longer (618μm and 634μm) than those of their parents (Col: 430μm and C24: 450μm) (Figure 4A); this phenomenon was also observed in F1 hybrids from C24 and Ws crosses (Figure 4B), suggesting the possibility of heterosis. However, this possible heterosis was not observed in F1 hybrids from crosses of An and Col (Figure 4C). Such differences, i.e. presence or absence of a heterotic effect, have previously been reported, depending on the parents used in the cross [18].

### Callose plugs continue to elongate in most ecotypes

**Figure 5:**
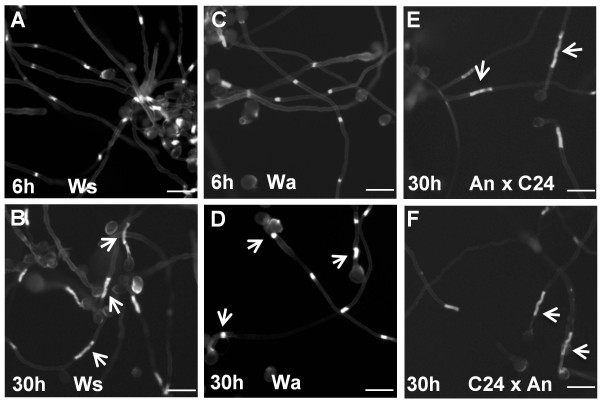
**Callose plugs continue to elongate in most ecotypes.** Ws pollen tubes after 6 h (**A**) or 30 h (**B**) germination. Callose plugs of Wa pollen tubes after 6 h (**C**) or 30 h (**D**) germination. (**E**) and (**F**) Callose plugs of pollen tubes of the F1s from reciprocal crosses between An and C24. Arrows indicate callose plugs. Bars = 50μm.

We noticed that the length of callose plugs in Col were longer when we had germinated pollen for a long time (30 hours) before staining with aniline blue. Therefore, we examined whether this phenomenon occurred in the other ecotypes. After 30 hours of germination, the C24, Van, Bay, Cvi, Ei, Nd, Nok, Got22, Est and Tsu and Ws ecotypes similarly had longer callose plugs (Figure 5B), but the callose plugs of An and Wa were not longer at 30 hours (Figure 5D). In ecotypes with longer callose plugs at 30 hours, it was mostly the first callose plug that had elongated (Figure 5B). To test if this phenotype was heritable, we examined F1 progeny of reciprocal crosses between An and C24. Pollen tubes of the F1s had longer callose plugs after 30h germination (Figure 5E and F), suggesting that the C24 phenotype was heritable and dominant. Callose plug elongation implies that callose synthase continues to be active at the callose deposition site, but it is not clear why ecotypes would vary for this parameter.

### Callose plug deposition is associated with pollen tube length

**Figure 6:**
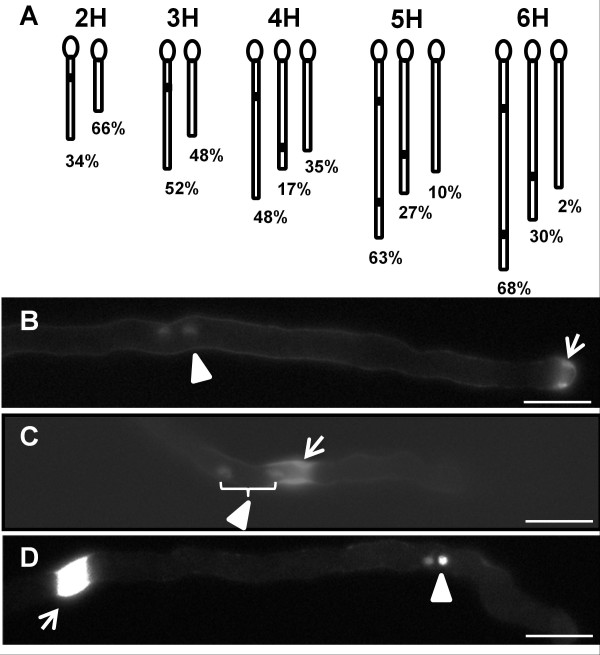
**Time course analysis of callose plug formation in the C24 ecotype.** (**A**). Schematic indicating pollen tubes with different callose plug patterns, e.g. the three pollen tubes shown at 4 hours pollen germination represent pollen tubes with a callose plug close to the pollen grain (left), with a callose plug far away from the pollen grain (middle) and one without a callose plug (right). The percentage represents the proportion of pollen tubes with each pattern. Tube lengths in the schematic represent the average pollen tube lengths in Table 1 but are not precisely to scale. (**B**) A forming callose plug near the pollen tube tip (arrow), sperm cells are behind it (arrowhead). Grain is to the left. (**C**) Sperm cells (arrowhead) passing through an incomplete callose plug (arrow). Grain is to the left. (**D**) A completely formed callose plug (arrow), sperm cells are in front (arrowhead). Grain is to the left. Bars = 30μm.

We were interested in determining where and when callose plugs are initiated. However, in the preceding experiments, most callose plugs were completely deposited and we were unable to directly observe callose plug initiation, because aniline blue staining and 4°C incubation halts pollen tube growth. We therefore used C24 and a time course experiment (Figure 6A; Table 1) to determine the approximate time that callose plugs started to form. After 2 hours of germination, 34% of the pollen tubes had a first callose plug close to the grain (first interval <150μm), whereas 66% of the pollen had shorter tubes (Figure 6A)) and had not yet formed a callose plug. After 3 hours of germination, more pollen tubes had formed a callose plug close to the grain, but about 50% of the tubes, although longer than at 2 hours, still had no callose plug. After 4 hours of germination, 65% of the pollen tubes had formed a first callose plug, including 48% with the first callose plug close to the grain and 17% with a first callose plug farther away from the grain, suggesting that most of the first callose plugs were formed within 4 hours of germination. Additionally, we noticed that the pattern of callose plugs with the first interval <150μm shifted to a pattern with the first interval ≥150μm between 3 to 4 hours germination, because the percentage of pollen tubes with the first interval <150μm decreased from 3h to 4h germination.

**Table 1:** Average length of pollen tubes with and without callose plugs in C24

	**2h**	**3h**	**4h**	**5h**	**6h**
tube lengths with callose plug	242±11 **	312±12**	391±11**	445±14**	485±13 **
tube length without callose plug	139±6	180±8	234±8	280±7	310±7
tube lengths with first interval <150um	242± 11	312±12	421±11 *	490±11**	533±17**
tube lengths with first interval ≥150um			362±11	400±11	437±13

Data are shown with mean(μm)±SEM. Significance was determined by an unpaired *t* test, *P<0.05, **P<0.01.

To determine where the first callose plugs form, we selected tubes that were forming callose plugs (2–5 hours germination), then determined the position where they started to form. Nearly all (93%) of the forming callose plugs were found near the pollen tube tip (Figure 6B), indicating that callose plug deposition started near pollen tube tip. This raised a question about the relationship between callose plug position and the position of the sperm cells during pollen tube growth. We therefore used a C24 line in which a *pDUO1*::*NLSmRFP* construct was present, and carried out a time course of pollen germination. The *DUO1* promoter is expressed in sperm cells [19] and thus the *pDUO1*::*NLSmRFP* reporter can be used to image sperm cell nuclei. The DAPI filter set used to visualize staining with aniline blue overlaps with the mRFP channel, so that we could see both the sperm nuclei and the callose plugs. Some callose plugs (including forming and completely formed callose plugs) were proximal to the position of the sperm (Figure 6B), and some callose plugs were distal to the position of the sperm (Figure 6D). The time course showed that around 30% of the sperm cells were behind the callose plug at each time point (Table 2). However, in movies generated from the images (e.g., Additional file 1; Figure 6C), sperm cells were seen passing through callose plugs in pollen tubes that had not yet ceased growth after staining with aniline blue. Therefore, it is plausible that the 30% sperm cells observed behind callose plugs were in tubes in which the aniline blue arrested growth before the sperm cells passed through the callose plug. It was previously reported in tobacco that vegetative and generative cells could be trapped behind callose plugs but could squeeze through a forming callose plug [20].

**Table 2:** Time course analysis to determine the relationship between sperm cell position and callose plugs

**Pollen germination hours**	**A**	**B**	**B/A**
3	27	7	0.26
4	34	9	0.26
5	66	23	0.35
6	128	36	0.28

A represents the total number of sperm cells investigated.

B represents the number of sperm cells behind the callose plug.

B/A represents the proportion of sperm cells behind the callose plug.

As many pollen tubes of the C24 ecotype did not have callose plugs after 2 or 3 hours germination, we were able to use C24 to investigate whether callose plug deposition correlated with pollen tube length, without any ecotype effect. The time course analysis (Table 1) was used to compare the lengths of pollen tubes with or without callose plugs. The average tube length of pollen tubes without a callose plug was significantly shorter than those with a callose plug. Additionally, after 4 hours of germination, the average length of tubes with the first callose plug close to the grain (i.e. first interval <150μm) was significantly longer than tubes with the first callose plug far away from the grain (first interval ≥150μm). We carried out correlation analyses with the 4, 5 and 6 hour time points; the first interval length and pollen tube length were significantly correlated at 5 hours (*r* = −0.235, *P*<0.05) and at 6 hours (*r* = −0.242, *P*<0.05), but not at 4 hours. Perhaps at the 4 hour time point there was still residual variability due to different times of pollen tube initiation.

### Variation of callose plug deposition in tomato species

**Figure 7:**
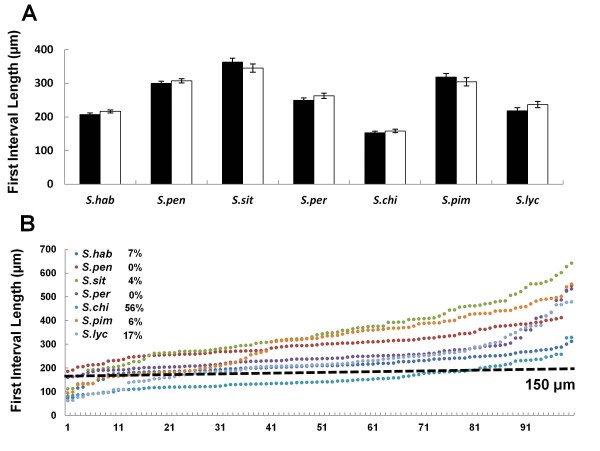
**Variation in first callose plug position in seven tomato species.** (**A**) Average length of the first interval in two independent experiments (black and white bars). An unpaired *t* test was performed between two independent experiments. Error bars are SEM, *P*< 0.5. (**B**) Distribution of first interval lengths. 150μm was used as a criterion to group first interval lengths. The percentages represent the proportion of pollen tubes with the first interval <150μm. S.hab: *S. habrochaites*; S.pen: *S. pennellii*; S.sit: *S. sitiens*; S.per: *S. peruvianum*; S.chi: *S. chilense*; S.pim: *S. pimpinellifolium*; S.lyc: *S. lycopersicum.*

**Figure 8:**
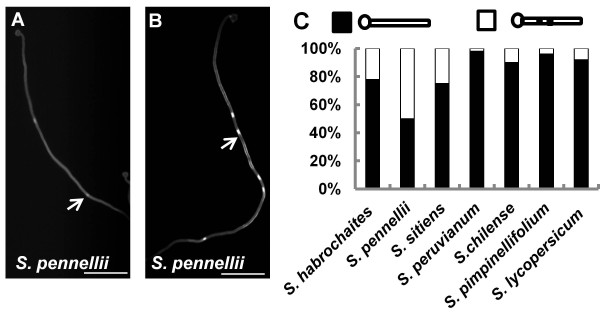
**Variation in second callose plug position in tomato species.** (**A**) A pollen tube with the second callose plug (arrow) far away from the first callose plug. (**B**) A pollen tube with the second callose plug (arrow) close to the first callose plug. (**C**) The proportions of second callose plug position pattern. Black represents tubes with the second callose plug far away the first callose plug; White represents tubes with the second callose plug close to the first callose plug. Bars in (**A**) and (**B**) = 50μm.

To determine if variation in the first callose plug position occurred in other species, we measured the first and second intervals and pollen tube lengths in seven different tomato species. The pollen germination method described in [8] for *Solanum lycopersicum* worked well for the other six species, and an unpaired *t* test showed that the average first intervals of 50 randomly selected healthy pollen tubes from two individual experiments were reproducible (Figure 7A). The average length of the first interval varied from 153 μm to 363 μm among the 7 species (Figure 7A). The two individual experiments (total of 100 pollen tubes) were pooled and used to determine the distribution of the first callose plug position. The same grouping criterion for first interval length used for *Arabidopsis* (Figure 1C) was used. Only *S. chilense* had a large portion (56%) of pollen tubes with the first interval length <150μm, suggesting that *S. chilense* had two patterns for the first callose plug position. The proportions of pollen tubes with the first interval <150μm in the other species were smaller than 20%, suggesting that those species mostly had one pattern for the first callose plug position. Although most intervals in those species were grouped into a single pattern, variations were observed (Figure 7B). Thus variation of the first callose position among different species or in one species also exists in tomato. We also noticed that *S. pennellii* showed two patterns for the second callose plug position: one close to first callose plug (Figure 8A) and another farther away (Figure 8B). We therefore checked all 7 tomato species; all had pollen tubes where the second callose plug position was far away from the first one, but *S. habrochaites*, *S. pennellii* and *S. sitiens* also showed a pattern where 22%, 50% or 25%, respectively, of the second callose plugs were near the first one. The phenomenon of callose plug elongation (Figure 5) was not observed in any of the tomato species after prolonged (30 hour) germination.

## Conclusions

Our studies show that all but one of the *Arabidopsis* ecotypes tested formed pollen tubes during in vitro germination. We showed that the position of the first callose plug varies among *Arabidopsis* ecotypes and in some cases within one ecotype. The callose plug deposition patterns were heritable; having two patterns of callose plug within an ecotype is dominant. Callose plug deposition correlated with pollen tube length and pollen tube lengths in F1 hybrids sometimes exceeded those of the parents. Variation in callose plug deposition was also seen in species of tomato. These assays were all carried out in vitro, and it is important to acknowledge that there might be differences in the parameters we measured when pollen tubes grow in the pistil, because of potential influences of the female tissue. Although the significance of these callose plug deposition differences are not known, these findings lay a foundation for mapping genes that regulate callose plug deposition or those that determine pollen tube lengths or growth rate.

## Methods

### Plant materials and growth conditions

Fourteen ecotypes of *Arabidopsis thaliana* (Col-0, Ws-0, C24, Est-1, Shahdara, Van-1, Bay-0, Tsu-1, Cvi-0, Nok-0, Ei-2, An-1, Nd-1, Wa-1, and Got-22) were used. All of the ecotypes except Col-0 were provided by Brian Staskawicz’s lab, UC Berkeley; they were originally obtained from NASC and the accession numbers are available there ( http://arabidopsis.info/). All Arabidopsis plants were grown in the greenhouse in a 4:1:1 mix of Fafard 4P: perlite: vermiculite under an 18-h light/6-h dark cycle at 21°C. Flowers of seven tomato species (*Solanum lycopersicum*, *Solanum pimpinellifolium*, *Solanum chilense*, *Solanum peruvianum Solanum sitiens*, *Solanum pennellii*, *Solanum habrochaites*) were obtained from plants grown in UC-Davis greenhouses.

### Pollen germination and aniline blue staining

*Arabidopsis* in vitro pollen tube germination was carried out as described in [15]. After 6h or 30h pollen germination, decolorized aniline blue was added and then the slides were immediately transferred to 4°C. A DAPI filter was used to observe callose plug and sperm cells. To observe the movement of sperm cells in the presence of aniline blue, we captured images, using a 40× objective, immediately after adding decolorized aniline blue (without transferring slides to 4°C). Tomato pollen germination followed the protocol in [8] . Decolorized aniline blue staining was as described in [21].

### Microscopic imaging and software for measuring pollen tube lengths and statistical analyses

Microscopic imaging was performed using an Axiovert microscope (Zeiss). Images were captured using a Spot digital camera (Diagnostic Instruments; http://www.diaginc.com/). Distances between a callose plug and the pollen grain pore were measured using Image J software ( http://rsbweb.nih.gov/ij/) [22]. The unaired *t* test, correlation analysis, analysis of variance (ANOVA) and multiple comparisons with least significant difference (LSD) were performed using SPSS 10.0 software (SPSS Inc., Chicago, IL, USA).

## Competing interests

The authors declare that they have no competing interests.

## Authors' contributions

PQ and SM designed the study, PQ, DT and AS performed the experiments, PQ and SM analyzed the data and wrote the manuscript. All authors read and approved the final manuscript.

## Supplementary Material

Additional file 1 Movement of sperm in pollen tube with a forming callose plug. Click here for file
